# Long-Term Monitoring of Everolimus Concentrations in Routine Clinical Practice: Evaluation of Patient Percentiles as Quality Indicator and Impact of Analytical Method Change

**DOI:** 10.3390/ph19091412

**Published:** 2026-09-07

**Authors:** Anders O. Larsson, Anna-Karin Hamberg, Mats B. Eriksson

**Affiliations:** 1 Department of Medical Sciences, Clinical Chemistry and Pharmacology, Uppsala University, SE-751 85 Uppsala, Sweden; anna-karin.hamberg@akademiska.se; 2 Department of Surgical Sciences, Anaesthesiology and Intensive Care, Uppsala University, SE-751 85 Uppsala, Sweden; mats.b.eriksson@uu.se; 3 NOVA Medical School, New University of Lisbon, 1169-056 Lisbon, Portugal

**Keywords:** everolimus, therapeutic drug monitoring, patient medians, patient percentiles, quality indicator, method comparison, immunoassay, transplantation, laboratory quality assurance

## Abstract

**Background**: Everolimus, an mTOR inhibitor used in oncology and organ transplantation, requires therapeutic drug monitoring due to the narrow therapeutic range of everolimus and substantial pharmacokinetic variation. Patient-derived data can function as a supplementary quality indicator for laboratory methods by providing real-world information on analytical performance. **Methods**: All routine whole-blood everolimus measurements performed at Akademiska University Hospital, Uppsala, Sweden, 2012 to 2025 were retrospectively evaluated. In total, 9,089 results were included. Annual and monthly concentration distributions were assessed using the 10th, 25th, 50th, 75th, and 90th percentiles. Long-term trends, sex differences, seasonal variation, and the impact of a method change from Architect (Abbott/Microgenics) to Cobas Pro e 801 (Roche) in 2021 were investigated. **Results**: Of 9089 results, 3335 were from females and 5754 from males. Concentration distributions were similar between sexes, with median concentrations of 4.47 and 4.33 µg/L, respectively; the overall median was 4.37 µg/L. Annual testing volumes remained stable. Median concentrations gradually declined from 5.22 µg/L in 2012 to approximately 4.0 µg/L in 2019–2020. Following introduction of the Cobas assay, patient concentration distributions shifted upward; compared with 2020, the 10th percentile increased by 45%, the median by 21%, and the 90th percentile by 11%. External quality assessment and method-comparison studies supported an analytical explanation. **Conclusions**: Everolimus concentration distributions were stable over time and showed no meaningful sex-related or seasonal variation. Patient percentile monitoring detected a clear analytical shift after method implementation and may serve as a practical supplementary quality indicator for TDM assays.

## 1. Introduction

Everolimus is a mammalian target of rapamycin (mTOR) inhibitor and an immunosuppressive agent widely used in solid organ transplantation, particularly kidney and liver transplantation, to prevent allograft rejection and facilitate calcineurin inhibitor minimization strategies [1,2]. In addition to its role in transplantation, everolimus is used in the treatment of several malignancies and selected disorders characterized by dysregulated cellular proliferation. Its immunosuppressive effects are mediated through inhibition of lymphocyte proliferation and progression of the cell cycle, key processes involved in adaptive immune responses and graft rejection [3]. 

Everolimus exerts its pharmacological action by binding to the intracellular immunophilin FK506-binding protein 12 (FKBP12), forming a drug–protein complex that inhibits the mTOR complex 1 (mTORC1) signaling pathway [4,5]. Inhibition of mTOR blocks downstream signaling involved in protein synthesis, cell growth, and cellular proliferation, thereby suppressing the proliferative responses of activated T and B lymphocytes to interleukin-2 and other growth-promoting cytokines. Unlike calcineurin inhibitors, everolimus acts downstream of cytokine receptor signaling and inhibits lymphocyte proliferation rather than cytokine production. 

Because everolimus has a relatively narrow therapeutic window, therapeutic drug monitoring (TDM) is routinely recommended for transplant recipients to optimize immunosuppressive efficacy while minimizing toxicity [6,7]. Excessive exposure may lead to adverse effects including dyslipidemia, proteinuria, impaired wound healing, stomatitis, hematological abnormalities, and increased susceptibility to infection, whereas insufficient exposure is associated with an increased risk of acute rejection and graft dysfunction. Consequently, TDM has become an integral component of clinical management in patients receiving everolimus-based immunosuppressive regimens. 

TDM of everolimus is typically performed using whole-blood trough concentrations (C0) [1,8]. Target concentration ranges vary according to transplant type, time after transplantation, concomitant immunosuppressive therapy, and the clinical objectives of treatment. Individualized dose adjustments based on measured concentrations are particularly important during the early post-transplant period, following dose modifications, and in the presence of factors that may alter drug disposition. 

Everolimus exhibits substantial interindividual pharmacokinetic variability. The drug is extensively metabolized by cytochrome P450 3A (primarily CYP3A4 and CYP3A5) and is a substrate of the efflux transporter P-glycoprotein [9,10]. Variability in gastrointestinal absorption, hepatic metabolism, drug–drug interactions, genetic factors, and patient-specific characteristics contributes substantially to interindividual differences in systemic exposure. These pharmacokinetic characteristics make fixed-dose approaches unreliable and underscore the importance of routine TDM in clinical practice. 

Given these analytical and pharmacokinetic challenges, this study aimed to evaluate long-term trends in patient-based median everolimus concentrations and assess potential seasonal variation [7,11,12]. The use of patient medians as a continuous quality indicator may provide additional insight into analytical stability and clinical practice patterns, particularly in settings where traditional quality control procedures are constrained by commutability limitations or restricted access to control materials.

We hypothesized that patient-derived percentile distributions would remain stable over time unless affected by a significant analytical change. A secondary hypothesis was that no clinically significant sex-related or seasonal variation would be observed in everolimus concentrations measured in routine clinical practice.

## 2. Results

### 2.1. Changes in Number of Reported Everolimus Results over Time

During the study period from 1 January 2012 to 31 December 2025, a total of 9089 everolimus results were reported (3335 results for females and 5754 results for males). The percentiles for females and males showed no major differences (Table 1). 

**Figure 1:**
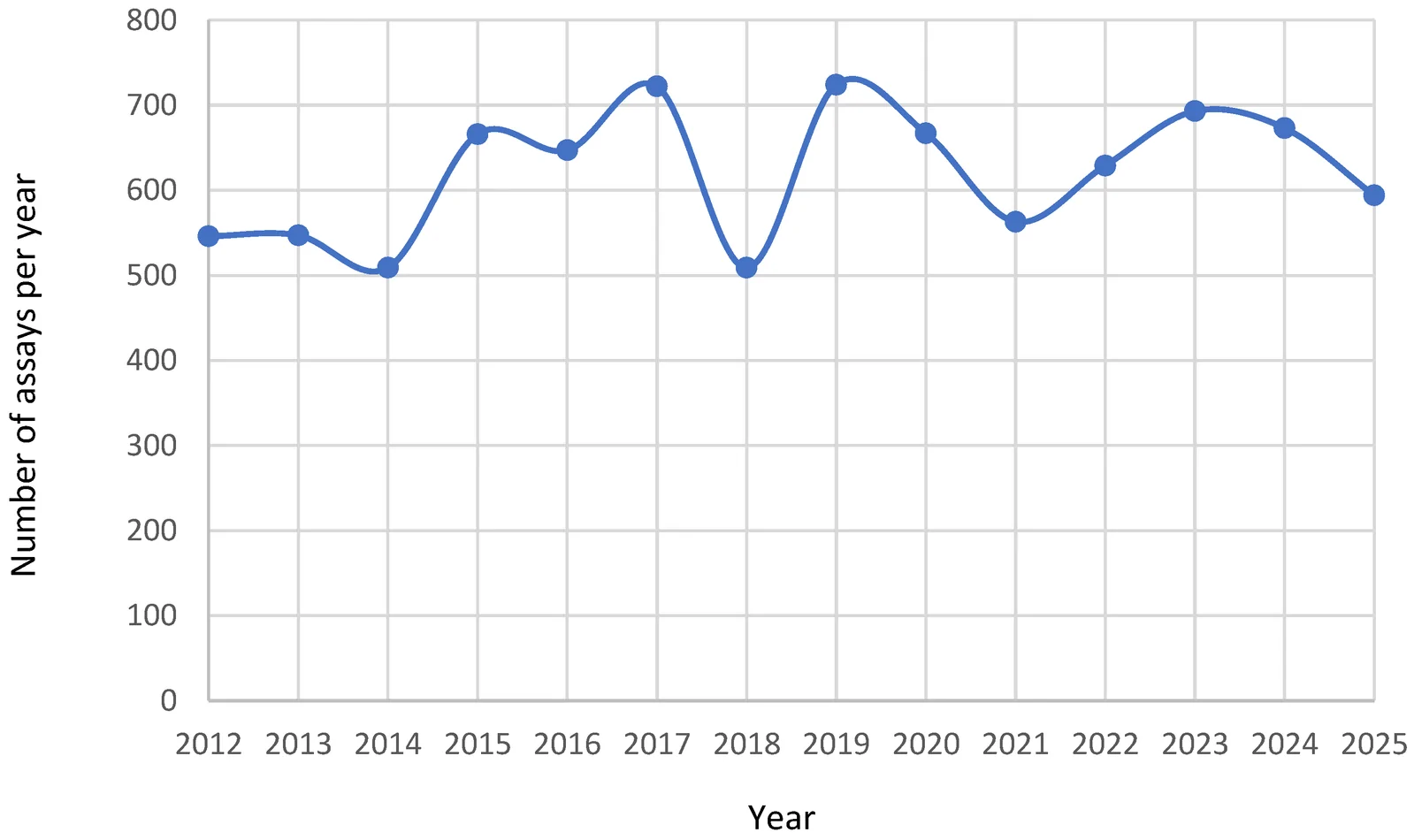
Graphical presentation of the number of annual everolimus measurements from 1 January 2012 to 31 December 2025.

The number of everolimus results per year over the 14-year study period was fairly stable, varying between 509 and 724 results per year (Figure 1).

### 2.2. Changes in Reported Everolimus Results over Time 

**Figure 2:**
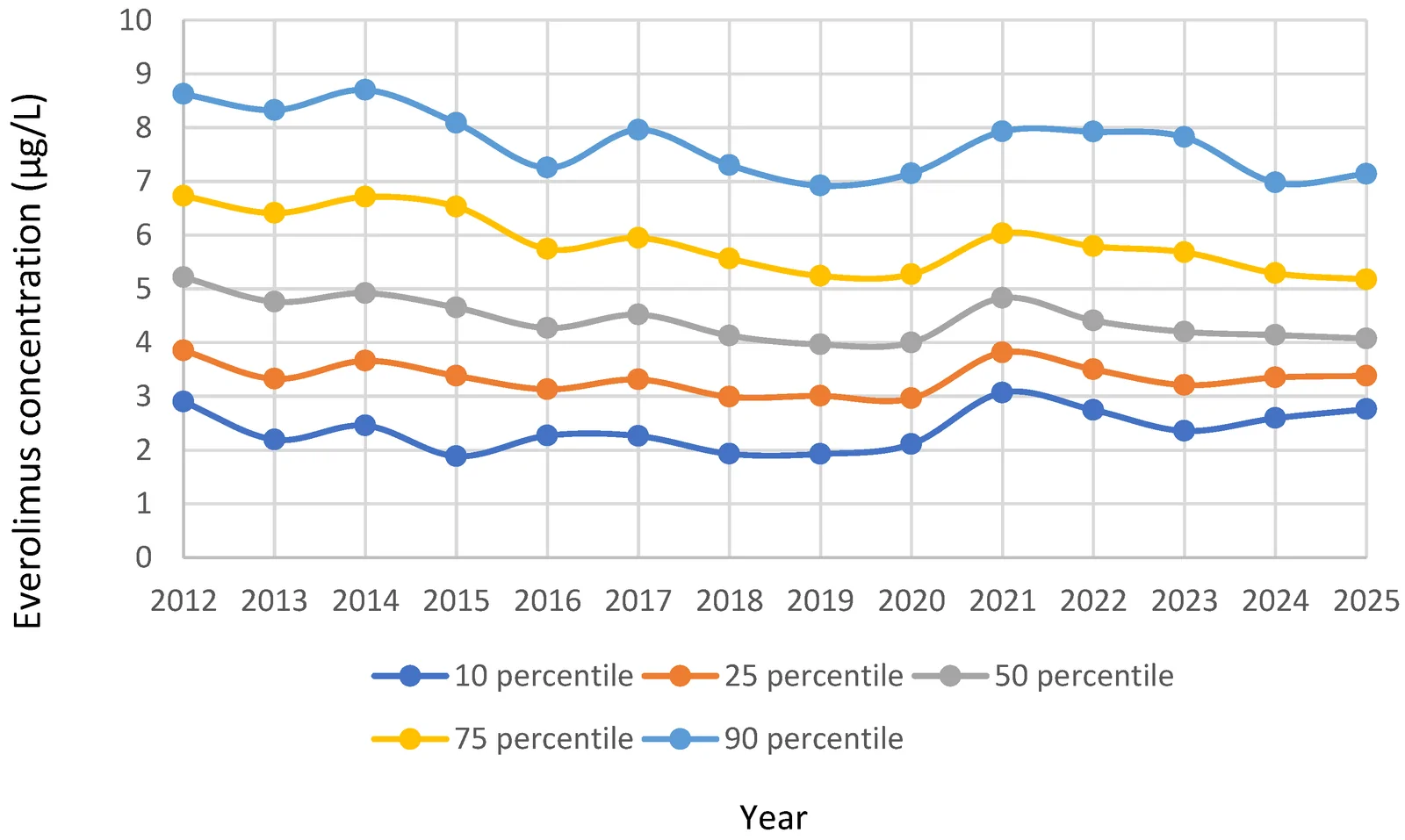
Graphical presentation of 10th, 25th, 50th, 75th, and 90th percentiles of everolimus concentrations (µg/L) in whole blood from 1 January 2012 to 31 December 2025.

The median everolimus values for the whole study period were 4.47 µg/L for females and 4.33 µg/L for males (Mann–Whitney U-test *p* = 0.0019). There was a slow decline for the higher percentiles over the years, interrupted by the method change in 2021. The method change from Architect to Cobas in 2021 resulted in increased median everolimus results by 45% for the 10th percentile, 21% for the 50th percentile and 11% for the 90th percentile (Figure 2).

### 2.3. Seasonal Variation in Everolimus Results

**Figure 3:**
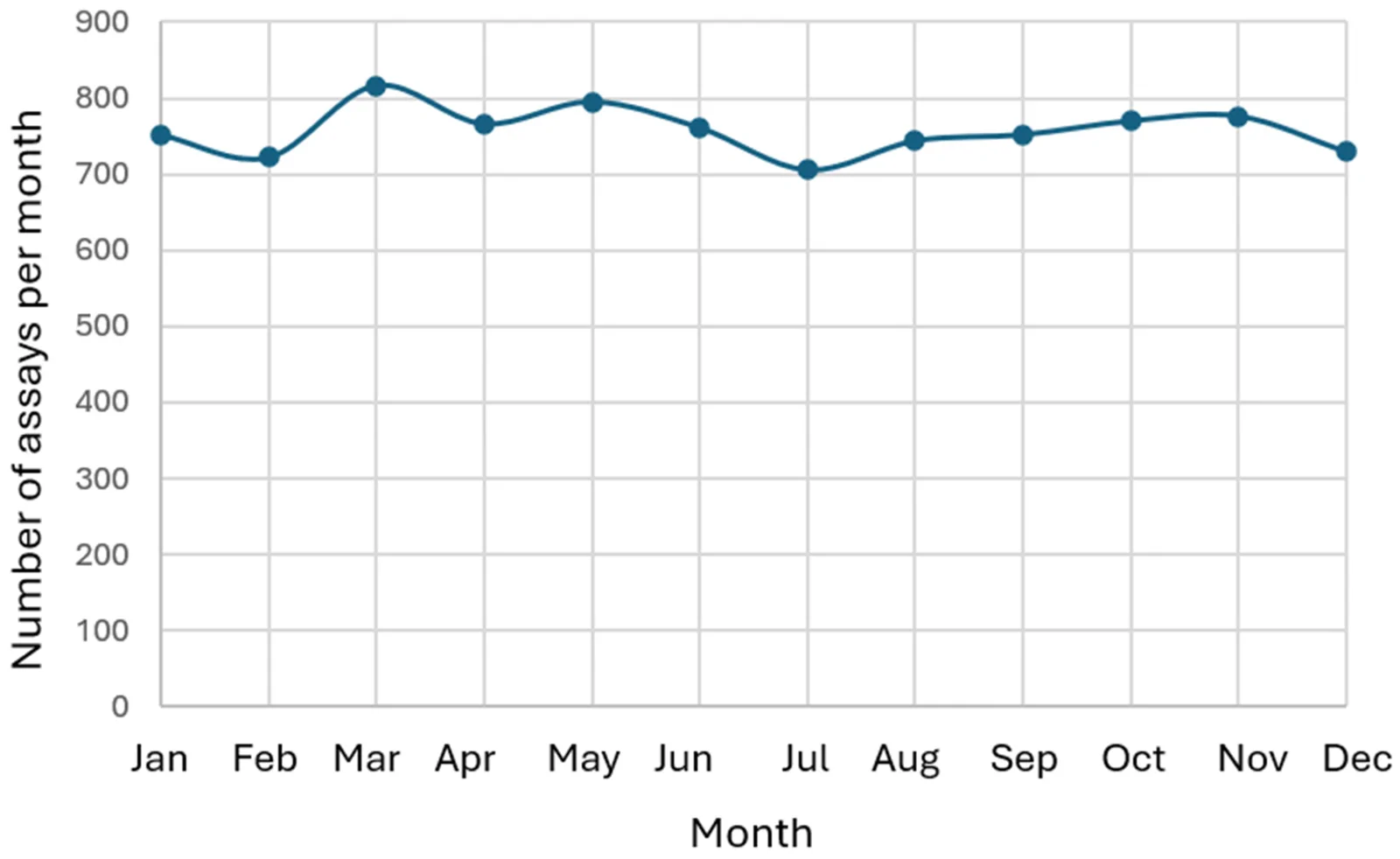
Graphical presentation of everolimus measurements per month.

**Figure 4:**
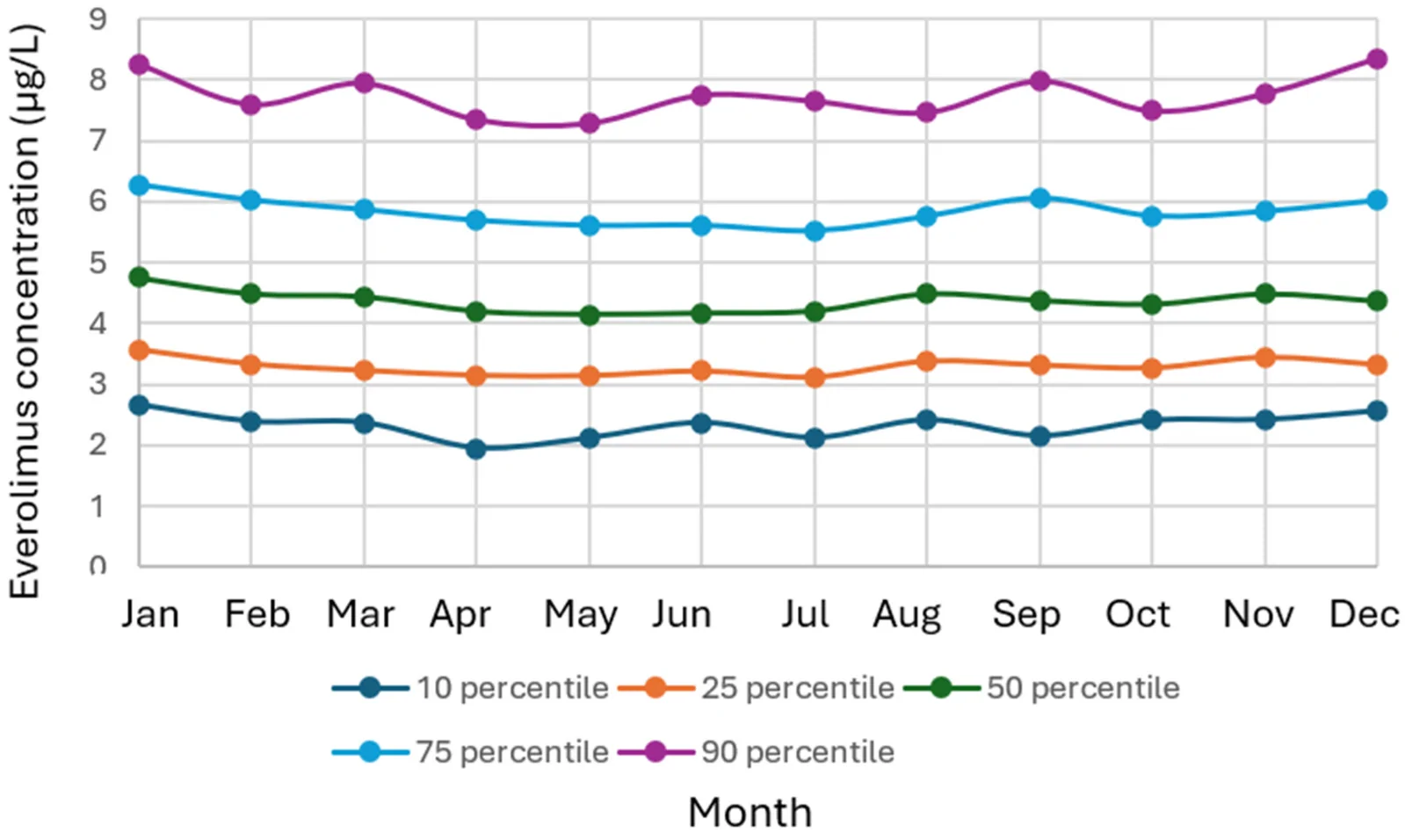
Monthly variation in everolimus measurements (µg/L), presented as percentiles.

The total number of reported everolimus results for each month was relatively stable during the study period, with the highest number of results reported in March (816) and the lowest in July (706) (Figure 3). The percentiles showed no major monthly variation (Figure 4). However, the 90th percentile showed slightly larger variation than the lower percentiles.

The 50th percentile varied between 4.14 and 4.76 for the entire study period. Monthly variation assessed using the Kruskal–Wallis test showed a significant variation (Chi-Square = 47.16415, df = 11, *p* < 0.0001).

**Figure 5:**
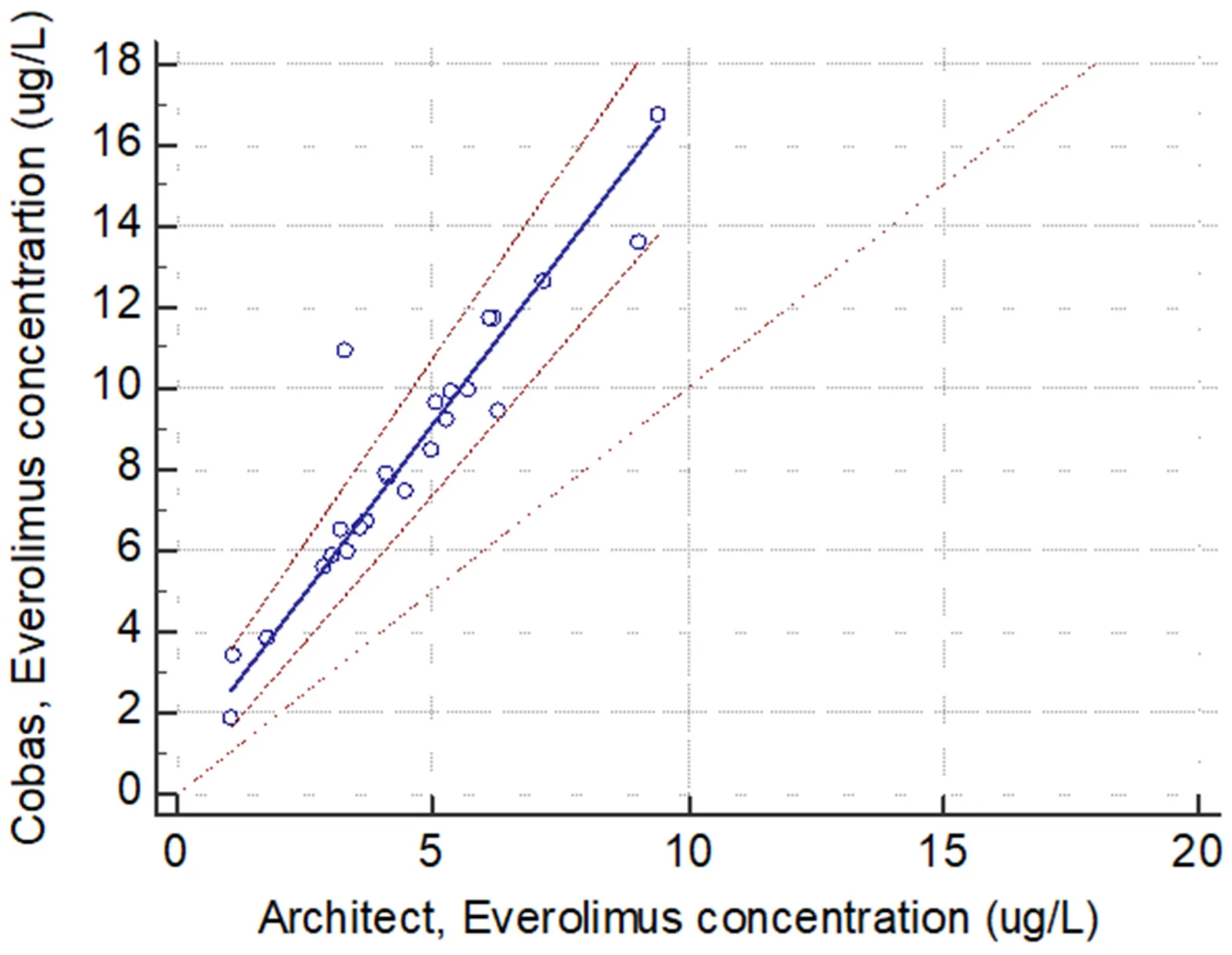
Comparison between everolimus concentrations measured by the Architect immunoassay (x-axis) and the Cobas immunoassay (y-axis). Each circle represents the result from a single patient sample analyzed by both methods (*n* = 24). The solid blue line is the Passing–Bablok linear regression line, describing the relationship between the two methods, while the red lines represent the 95% confidence interval around the regression. The dotted diagonal line is the line of identity (y = x), indicating perfect agreement between the two methods.

Method validation was performed when the method was changed from the Architect platform to the Cobas platform, comparing results for patient samples and external quality assurance samples analyzed with both the Architect and Cobas methods, and showed clearly higher values with the Cobas method than with Architect (Figure 5). This is in agreement with the increased patient values observed between 2020 and 2021. The Passing–Bablok regression equation for the method comparison was y = 1.65x + 0.835. The 95% confidence interval for the slope was 1.45–1.82 and the 95% confidence interval for the intercept was 0.112–1.651. The method comparison included *n* = 24 paired samples and yielded a correlation coefficient of r = 0.94.

## 3. Discussion

In this study, we evaluated trends in everolimus concentrations measured in routine clinical practice over a 14-year period. A total of 9089 results were included, providing a unique opportunity to assess temporal changes in patient distributions and to explore the utility of patient percentiles as a quality indicator for therapeutic drug monitoring (TDM). The principal findings were that (i) everolimus concentrations were generally stable over time, (ii) no clinically meaningful seasonal variation was observed, (iii) no major differences were found between male and female patients, and (iv) a clear shift in concentration distributions occurred following the change of analytical method from Architect to Cobas in 2021.

The overall distribution of everolimus concentrations was stable throughout the study period. The median concentration for the entire population was 4.37 µg/L, with relatively narrow variation between annual medians. This finding suggests that clinical dosing practices and treatment targets remained largely consistent despite changes in oncological practice [13,14] and transplant protocols in the patient population over time. Since everolimus dosage is routinely adjusted on the basis of measured trough concentrations, a stable population median likely reflects both consistent clinical practice and satisfactory analytical performance.

No clinically significant differences were observed between male and female patients. Females demonstrated slightly higher concentrations across all percentiles, but the differences were small and unlikely to be of clinical importance. This observation is consistent with current dosing approaches for everolimus, which are based primarily on concentration-guided dose adjustment rather than sex-specific dosing algorithms. Any sex-related differences in pharmacokinetics may therefore be mitigated through routine TDM and subsequent dose individualization.

One of the most important findings was the marked increase in patient concentrations following implementation of the Roche Cobas method in January 2021. The increase was evident across the entire concentration distribution but was particularly pronounced in the lower percentiles, with the 10th percentile increasing by approximately 45%, compared with 21% for the median and 11% for the 90th percentile. This pattern strongly suggests a systematic analytical difference between the methods rather than a true change in patient exposure [15,16,17].

The observed increase corresponded closely with the findings of the method validation studies, which demonstrated consistently higher results using the Cobas assay compared with the Architect assay. Furthermore, external quality assurance samples analyzed using both methods showed similar differences. Together, these observations indicate that the shift in patient percentiles was primarily attributable to the analytical method change. The results illustrate how continuous monitoring of patient median values can rapidly identify systematic shifts following implementation of new analytical methods and provide a complementary tool to conventional validation procedures. Although the Cobas method produced higher everolimus concentrations, a gradual decline in concentrations was observed after 2021. This likely reflects clinical dose adjustments following implementation of the new assay. Clinicians were informed that Cobas values were approximately 30% higher, and dose reductions were expected to maintain target trough concentrations.

Interestingly, the increase observed in routine patient data appeared larger than the approximately 30% increase identified during method validation, particularly at lower concentration ranges. This may reflect concentration-dependent differences between the assays, changes in calibration characteristics, or differential cross-reactivity with everolimus metabolites. Immunoassays for immunosuppressive drugs are known to be influenced by antibody specificity and metabolite recognition, which may lead to differences between analytical platforms [7,8]. The study by Shipkova et al. showed 34% higher values with a Roche method compared to LC-MS/MS [8], which is in agreement with our findings. The disproportionately larger increase in low-concentration samples suggests that the relationship between the two methods may not be completely linear throughout the measuring range. The disproportionately larger increase at lower percentiles may have clinical implications, particularly for oncology patients who often have lower target ranges. Concentration-dependent bias could lead to overestimation of exposure in low-dose regimens, underscoring the importance of method-specific interpretation. Because no LC MS/MS reference method was available during the method transition, the magnitude and nature of the observed bias cannot be definitively attributed to immunoassay characteristics alone. Future studies including LC MS/MS comparison would strengthen conclusions regarding assay-related bias. Although the 45% increase at the 10th percentile primarily reflects analytical bias introduced by the Cobas method, such a shift may have clinical implications. Low range everolimus concentrations are often used to guide dose escalation, particularly in oncology patients and in transplant recipients receiving combination immunosuppression. A systematic upward shift at the lower end of the distribution may lead to overestimation of exposure and could delay necessary dose increases. Because clinical outcome data were not available, the magnitude of this impact cannot be quantified, and these considerations should be interpreted cautiously.

The annual percentile data also demonstrated a gradual decline in median everolimus concentrations between approximately 2012 and 2020. The median decreased from 5.22 µg/L in 2012 to approximately 4.0 µg/L during 2019 to 2020. Several explanations may account for this trend. Clinical practice has increasingly favored lower exposure targets when everolimus is combined with tacrolimus or other immunosuppressive agents to reduce toxicity while maintaining efficacy. Changes in patient selection, transplant protocols, oncological practice, and target concentration recommendations may therefore have contributed to the gradual reduction observed before introduction of the Cobas method. Alternative explanations include increased use of tacrolimus–everolimus combination therapy, evolving toxicity avoidance strategies, and changes in clinical guidelines. These factors may have contributed to the decline in median concentrations. Because patient-level covariates such as transplant type, indication, and concomitant immunosuppressive therapy were unavailable, the gradual decline in median concentrations between 2012 and 2020 may be entirely attributable to changes in case mix. Without these data, no firm conclusions can be drawn regarding changes in clinical practice or dosing strategies. Recommended therapeutic ranges for everolimus have evolved over the study period, particularly in transplant medicine where lower target concentrations (3–6 µg/L) have increasingly been adopted when everolimus is combined with tacrolimus. Earlier protocols often targeted higher ranges (4–8 µg/L). These evolving recommendations may have contributed to the gradual decline in median concentrations observed between 2012 and 2020. Everolimus target concentrations differ depending on concomitant immunosuppressive therapy. Increasing use of tacrolimus–everolimus combination regimens and reduced reliance on cyclosporine may have influenced population-level concentration distributions. Because regimen data were not available, this represents an important confounder.

A previous study has reported seasonal variation in CYP3A activity [18]. As everolimus is metabolized by cytochrome P450 3A, this could in theory induce a seasonal variation in everolimus concentrations. Seasonal variation in the present study was minimal. Both the number of samples and the concentration percentiles remained relatively stable throughout the calendar year. Monthly variation for the concentration reached statistical significance (*p* < 0.0001), but the absolute differences in median concentrations were small (<0.3 µg/L) and well within expected biological and analytical variation. Thus, the variation is statistically detectable due to the large sample size but not clinically meaningful. Although the 90th percentile showed somewhat greater month-to-month variation, in both absolute and relative numbers, than the lower percentiles, the magnitude of these fluctuations was small and unlikely to represent clinically meaningful changes. The absence of substantial seasonal variation suggests that environmental factors such as sunlight exposure, dietary changes, or seasonal variations in concomitant medications have limited impact on everolimus concentrations at a population level. From a quality assurance perspective, the lack of systematic monthly variation supports the analytical robustness of the assay throughout the year. 

The present study highlights the value of patient percentile monitoring as a form of indirect analytical surveillance [19,20]. Traditional quality control procedures rely on internal quality control materials and external quality assessment samples, both of which may be affected by commutability limitations [21]. Analysis of large patient datasets provides an additional real-world perspective on assay performance and may reveal systematic shifts that are not easily detected by conventional quality assurance programs. The abrupt shift observed in 2021 demonstrates the sensitivity of this approach. Monitoring patient medians and percentile distributions may therefore serve as a useful complement to established quality management systems, particularly for assays used in therapeutic drug monitoring [22,23,24,25].

A number of limitations should be acknowledged. First, this was a retrospective study based on routine laboratory data, and no clinical information regarding transplantation type, indication for therapy, dosing regimen, sampling time, concomitant medications, or treatment targets was available. Consequently, observed differences cannot be directly linked to clinical decision-making. Second, repeated samples from individual patients were included, meaning that the dataset reflects routine monitoring activity rather than unique patients. A sensitivity analysis using only the first everolimus measurement per patient per year produced trends consistent with the full dataset, indicating that repeated measurements did not materially bias the percentile distributions. Third, changes in patient demographics and prescribing practices over time may have influenced the observed distributions. Several clinical variables known to influence everolimus exposure, including hepatic function, CYP3A/P glycoprotein-interacting medications, body weight, and pharmacogenetic background, were not available. Their absence limits interpretation of temporal trends and represents a major limitation. No clinical outcome data (rejection, toxicity, dose adjustments) were available. This limits interpretation of the clinical significance of observed concentration trends and represents a major limitation. Finally, the study was performed at a single center and may therefore not fully reflect practice patterns at other institutions.

## 4. Materials and Methods

### 4.1. Samples

All reported results from whole-blood everolimus measurements submitted to the Department of Clinical Chemistry and Pharmacology, Akademiska University Hospital, Uppsala, Sweden, were included in the present study. Blood samples were collected in EDTA-containing tubes (BD Vacutainer^®^, ref. 367862; BD Vacutainer Systems, Plymouth, UK). The study covered the period from 1 January 2012 through 31 December 2025, during which 9089 everolimus results were reported. Data were retrieved from the laboratory information system according to the approved ethical protocol and were fully pseudonymized prior to analysis. Available variables comprised patient age, sex, date of sampling, and measured everolimus concentration. No information enabling patient identification was accessible to the investigators. The study was approved by the Regional Ethical Review Board at Uppsala University (Dnr 01-367). The original ethical approval from 2001 covers the study protocol and has been maintained since.

### 4.2. Instruments

Until December 2020, everolimus concentrations were measured on the Architect chemistry analyzer (Abbott Laboratories, Abbott Park, IL, USA) using immunoassay reagents supplied by Microgenics (0373852, Microgenics Corporation, Fremont, CA, USA). Calibration and internal quality control procedures were carried out in accordance with the manufacturer’s recommendations. External quality assessment was performed through UK NEQAS (Sheffield, UK) and was reviewed monthly. The analytical coefficient of variation (CV) was 4.2% at 3.5 µg/L, and 8.4% at 7.1 µg/L. 

In January 2021, everolimus analysis was moved to the Cobas Pro e 801 platform (Roche Diagnostics, Mannheim, Germany) using Roche Elecsys^®^ Everolimus reagents (Roche Diagnostics, Mannheim, Germany). Prior to implementation, the method underwent analytical validation, including comparison of patient specimens analyzed by both platforms, evaluation of analytical bias and imprecision, assessment of calibration performance, and verification of the reportable measuring range.

Everolimus concentrations were initially measured using the Quantitative Microsphere System (QMS^®^) Everolimus assay (Microgenics Corporation, Fremont, CA, USA) on an Architect c8000 analyzer (Abbott Laboratories, Abbott Park, IL, USA). The assay was a homogeneous particle-enhanced turbidimetric immunoassay developed for the quantitative determination of everolimus in whole blood. Prior to analysis, specimens underwent protein precipitation according to the manufacturer’s instructions. Because methanol is highly volatile, samples were processed immediately after methanol addition to minimize evaporation-related analytical errors. The assay was based on competition between everolimus present in the patient sample and everolimus-coated microparticles for binding sites on polyclonal anti-everolimus antibodies. In the absence of analyte, rapid agglutination of the coated microparticles occurred, resulting in an increase in absorbance. When everolimus was present in the sample, agglutination was inhibited in a concentration-dependent manner, producing a smaller increase in absorbance. Consequently, the measured absorbance was inversely proportional to the everolimus concentration. Absorbance was measured photometrically at 700 nm. Calibration was performed using the QMS Everolimus Calibrator Set, which was traceable to a validated liquid chromatography-tandem mass spectrometry (LC-MS/MS) reference method. The calibrator set consisted of six standards with concentrations of 0.0, 1.5, 3.0, 6.0, 12.0, and 20.0 µg/L. Calibrators were stored frozen below −15 °C until first use and remained stable for six weeks at 2 to 8 °C after thawing. Internal quality control was performed using a three-level QMS Everolimus Control Set with target concentrations of approximately 4, 8, and 16 µg/L. Controls were analyzed with each analytical run, and assigned values were verified during the implementation of new reagent lots.

The analytical measurement range was 0.0 to 20.0 µg/L. Samples with concentrations exceeding the highest calibrator were diluted with calibrator A (0.0 µg/L) or drug-free EDTA whole blood before the addition of methanol and the precipitation reagent. Final concentrations were corrected for the applied dilution factor. The results were reported in µg/L with one decimal place, and concentrations below 2.0 µg/L were reported as <2.0 µg/L.

Interference studies demonstrated less than 10% bias in the presence of bilirubin concentrations up to 1020 µmol/L, cholesterol concentrations up to 9.0 mmol/L, gamma globulin concentrations up to 120 g/L, rheumatoid factor concentrations up to 135 IU/mL, triglyceride concentrations up to 17.1 mmol/L, and hematocrit values up to 60%.

The Architect method was later replaced by the Cobas method.

For everolimus testing on the Cobas instrument, sample pretreatment was performed manually using the Elecsys ISD Sample Pretreatment reagent, which lysed blood cells, extracted everolimus, and precipitated most blood proteins. Following centrifugation, the supernatant was used for analysis. Everolimus concentrations were determined on the Cobas Pro e 801 analyzer (Roche Diagnostics, Mannheim, Germany) using the Elecsys Everolimus immunoassay. The assay employed a competitive immunometric principle with electrochemiluminescence detection. In the first incubation, everolimus in the pretreated sample competed with a ruthenium-labeled everolimus derivative for binding to a biotinylated monoclonal anti-everolimus antibody. In the second incubation, streptavidin-coated microparticles were added, allowing the immune complexes to bind to the solid phase. The reaction mixture was aspirated into the measuring cell, where microparticles were magnetically captured on the electrode surface. Unbound substances were removed, and application of voltage induced chemiluminescent emission, which was measured by a photomultiplier. Everolimus concentrations were calculated from a two-point calibration curve adjusted to a master curve traceable to gravimetrically prepared whole blood calibrators. The analytical measuring interval was 0.33–19.5 µg/L. Samples exceeding this range were manually diluted 1:2 with Diluent Universal, pretreated, and reanalyzed; final concentrations were corrected for the dilution factor. Internal quality control was performed at three concentration levels using whole blood control material, and external quality assessment was conducted six times per year using LGC immunosuppressive drug materials. The assay’s specificity was evaluated by the manufacturer, who reported no interference from common endogenous substances or medications within tested concentrations. Cross-reactivity with sirolimus and several everolimus metabolites was documented, and samples from patients recently treated with sirolimus required appropriate washout periods or LC MS/MS confirmation.

The results were reported in µg/L with one decimal between 0.5 and 19.9 µg/L and as integers ≥ 20 µg/L. Values < 0.5 µg/L were reported as below the quantification limit. All results were reviewed in relation to previous measurements and documented dose adjustments before release.

Method comparison studies demonstrated that the Roche assay produced approximately 30% higher everolimus concentrations than the previously used Architect method. To facilitate clinical interpretation during the transition period, all reported results were accompanied by a comment indicating that concentrations obtained with the new method were approximately 30% higher than those generated by the previous assay.

Following implementation of the Roche method, external quality assessment was conducted monthly using materials supplied by LGC Standards (Wesel, Germany). The total analytical CV was 4.3% at 7.7 µg/L, 2.6% at 12.9 µg/L and 5.6% at 24.4 µg/L. The laboratory reports the results in µg/L and the recommended interval for trough samples was 3–8 µg/L.

### 4.3. Statistical Calculations

The distribution of everolimus concentrations was evaluated using descriptive statistics. The 10th, 25th, 50th, 75th, and 90th percentiles were calculated for all reported everolimus results and used to assess longitudinal trends and variability over time. In addition, monthly percentile distributions were examined to identify potential seasonal variation. We evaluated the distribution of repeated measurements. No single patient contributed disproportionately to monthly or annual percentiles. Annual and monthly percentiles were calculated using all available observations. Repeated measurements from the same patient were retained because the objective was to evaluate routine clinical testing patterns and population-level monitoring performance. No exclusions were applied other than records with missing data required for analysis. Differences between male and female patients were evaluated using the Mann–Whitney U test. Monthly variation was assessed using the Kruskal–Wallis test. Statistical significance was defined as *p* < 0.05. Statistical calculations were performed using Microsoft Excel 365 (Microsoft Corporation, Redmond, WA, USA) and Statistica version 10 (TIBCO Software Inc., Palo Alto, CA, USA) for the Mann–Whitney U test and Kruskal–Wallis test.

## 5. Conclusion

In conclusion, long-term monitoring of everolimus concentrations demonstrated generally stable patient distributions. Although sex-related and monthly differences reached statistical significance, the absolute differences were small and not clinically meaningful. A pronounced shift in concentration percentiles was observed following transition from the Architect to the Cobas assay in 2021, consistent with method validation findings indicating higher results with the Cobas method. These findings support the use of patient percentile analysis as a valuable supplementary quality indicator capable of detecting analytical shifts and providing insight into long-term trends in therapeutic drug monitoring practice. 

## Figures and Tables

**Table 1:** 10th, 25th, 50th, 75th, and 90th percentiles of everolimus concentrations (µg/L) in whole blood by sex.

	Number	10th	25th	50th	75th	90th
Both sexes	9089	2.34	3.29	4.37	5.84	7.78
Females	3335	2.36	3.34	4.47	5.92	7.99
Males	5754	2.34	3.25	4.33	5.79	7.63

## Data Availability

The raw data supporting the conclusions of this article will be made available by the authors on request.
